# Elevated blood pressure and headache disorders in China – associations, under-treatment and implications for public health

**DOI:** 10.1186/s10194-015-0570-0

**Published:** 2015-10-05

**Authors:** Mianwang He, Shengyuan Yu, Ruozhuo Liu, Xiaosu Yang, Gang Zhao, Xiangyang Qiao, Jiachun Feng, Yannan Fang, Xiutang Cao, Timothy J. Steiner

**Affiliations:** Department of Neurology, Chinese PLA General Hospital, Fuxing Road 28, Haidian District, 100853 Beijing, China; Department of Neurology, Xiangya Hospital, Central-South University, Xiangya Road 87, 410008 Changsha, Hunan Province China; Department of Neurology, Xijing Hospital, The Fourth Military Medical University, Changle West Road 15, 710032 Xian, Shaanxi Province China; Department of Neurology, Affiliated Huashan Hospital of Fudan University, Urumqi Middle Road 12, 200040 Shanghai, China; Department of Neurology, The First Hospital of Jilin University, Xinmin Street 71, 130021 Changchun, Jilin Province China; Department of Neurology, The First Affiliated Hospital of Sun Yat-sen University, The Second Zhongshan Road 58, 510080 Guangzhou, Guangdong Province China; Department of Health and Economics, Chinese PLA General Hospital, Fuxing Road 28, Haidian District, 100853 Beijing, China; Department of Neuroscience, Norwegian University of Science and Technology, Trondheim, Norway

**Keywords:** Headache, Hypertension, Public health, Population-based survey, China, Global campaign against headache

## Abstract

**Background:**

Both hypertension (HTN) and headache disorders are highly prevalent worldwide. Our purpose, in a nationwide study of the Chinese general population, was to evaluate any association between primary headache disorders and elevated blood pressure (eBP). We could not collect data on antihypertensive therapy, but took the view that, whatever such therapy might be taken, eBP was a sign that it was failing to meet treatment needs. Therefore, as a secondary purpose, important from the public-health perspective, we would present the prevalence of eBP (treated or not) as indicative of unmet health-care need in China.

**Methods:**

This was a questionnaire-based nationwide cross-sectional door-to-door survey using cluster random-sampling, selecting one adult (18–65 years) per household. Headache was diagnosed by ICHD-II criteria and eBP as systolic blood pressure ≥140 mmHg and/or diastolic blood pressure ≥90 mmHg. Chi-squared test and multivariate logistic regression analysis were used to assess the strength and significance of associations. We set significance at *P* ≤ 0.05.

**Results:**

Of 5,041 survey participants (participation rate 94.1 %), 154 were excluded because of missing BP data, leaving 4,987 for analysis [mean age: 43.6 ± 12.8 years; male 2,532 (mean age: 43.4 ± 12.9 years); female 2,455 (mean age 43.9 ± 12.8 years)]. There were 466 participants with migraine, 535 with tension type headache (TTH) and 48 with all causes of headache on ≥15 days/month. The prevalence of eBP was 22.1 % (males 22.9 %, females 21.3 %). No associations of eBP with any of the headache disorders survived multivariate adjusted analysis. The demographic and anthropometric variables most strongly associated with eBP were higher age (AOR 3.7) and being overweight (AOR 2.4), seen in both genders. Less strong were male gender, lower educational level and urban habitation.

**Conclusions:**

We found no clear-cut associations between eBP and any headache disorder. The associations with demographic and anthropometric variables may have acted as confounders in past reports to the contrary. We did find an alarmingly high prevalence of eBP, recognizing that this signals substantial under-treatment in China of a serious condition, and therefore a major public-health concern.

## Background

Both hypertension (HTN) and headache disorders are highly prevalent worldwide. HTN is recognised as a global public-health problem affecting over one quarter of adults in the world [1]. It is one of the major risk factors for cardiovascular diseases (CVDs), from which an estimated 17.3 million people worldwide die each year, a number expected to rise to 23.3 million by 2030 [2]. Thereby, *inter alia*, it contributes substantially to the burden of non-communicable diseases [2], while over 80 % of these deaths occur in low- and middle-income countries [2]. Active headache disorders affect 46 % of adults in the world, with regional variations [3], and are the third leading cause of disability worldwide [4].

China, the most populous country in the world, appears to be no exception as far as HTN is concerned: a recent study reported an adult prevalence of26.6 % [5]. Headache disorders are somewhat less prevalent than the global mean: we found, in a nationwide population-based study, a 1-year prevalence of primary headache disorders of 23.8 %, of migraine 9.3 %, of tension-type headache(TTH) 10.8 %, and of all causes of headache occurring on ≥15 days/month 1.0 % [6]. The relatively low prevalence of migraine is reflected also in Japan [7] and Taiwan [8], and appears to be genetically determined. Even so, about a quarter of Chinese adults have a headache disorder, and a similar proportion have HTN.

The association between HTN and headache, especially primary headache, is controversial. Two studies have suggested there is no association [9, 10]. Using ambulatory blood pressure (BP) monitoring, two studies found no association between the occurrence of headache and BP variation in patients with mild-to-moderate hypertension [11, 12], and no variation of BP around episodes of TTH and migraine-like headache [11]. Some studies found inverse relationships: between HTN and migraine in men [13], between BP level and migraine in the elderly [14], between BP level and migraine [15], and between systolic BP and subsequent development of headache [16]. Other studies found direct relationships between headache and prevalence of HTN, including severe migraine, TTH and other headache [17], and migraine, TTH and “chronic daily headache” [18]. Clearly, consensus is lacking.

We investigated the prevalence of primary headache disorders and possibly associated factors in a large nationwide cross-sectional survey of the Chinese population [6, 19], as a project within the Global Campaign against Headache [20]. One of the variables of interest was BP. In the context of this headache-specific survey we could not collect information on any antihypertensive therapy being taken, and we could not and did not seek to assess the prevalence of HTN. Instead we aimed to establish the prevalence of elevated BP (eBP), and its association with headache. This was the first population-based study to do this in mainland China.

Additionally, we took the view that, whatever antihypertensive therapy might be taken, eBP was a sign that it was failing to meet treatment needs. Therefore, as a secondary but important purpose from the public-health perspective, we would present the prevalence of eBP (treated or not) as indicative of unmet health-care need in China.

## Methods

### Ethics

The study protocol was approved by the Chinese Ministry of Health and the ethics committee of the Chinese PLA General Hospital, Beijing, China.

### Study design

In 2009 to 2010, we performed a cross-sectional door-to-door survey of the adult population throughout China, obtaining a representative sample of the adult population using cluster random-sampling software according to the EPI method established by the World Health Organization [12]. These methods have been described in detail elsewhere [6, 19]. The enquiry employed a structured questionnaire developed by *Lifting The Burden* [20] for population-based studies, translated into Chinese from the English version and validated within the target population in a sub-study [19]. Demographic enquiry included age, gender, habitation, marital status, occupation and educational level. Headache diagnoses were based on International Classification of Headache Disorders 2nd edition (ICHD-II) criteria [21].

### Blood pressure measurements

BP was measured twice in the sitting position after rest for at least 15 minutes using a calibrated aneroid sphygmomanometer; the mean values of systolic (SBP) and diastolic blood pressure (DBP) were taken. We defined eBP as SBP ≥ 140 mmHg and/or DBP ≥ 90 mmHg [22].

### Anthropometric measurements

All body measurements used standard anthropometric protocols. Weight and height were recorded using a portable calibrated digital scale. Body mass index (BMI) was calculated as weight divided by square of height. We defined overweight as BMI ≥ 25.0 kg/m^2^ as described in our previous study [23].

### Statistical analysis

All demographic variables were categorized (see Table 1) and presented as percentages. Age was also expressed as means ± standard deviations (SDs). Prevalences of headache and of eBP were estimated as percentages with 95 % confidence intervals (CIs). We used independent chi-squared tests for significance of differences in primary headache prevalence between people with eBP and those without, regarding *P* ≤ 0.05 as significant. We then calculated adjusted odds ratios (AORs) with 95 % CIs by multivariate logistic regression taking into account the following variables: age, gender, habitation, marital status, occupation, educational level, body weight. We took the group with normal BP as the reference. We used similar methods to examine possible associations between eBP and demographic and anthropometric variables.

**Table 1:** Demographic, anthropometric and blood pressure data of the sample (*N* = 4,987)

Variable	Category	Number	Percent
Gender	Male	2,532	50.8
	Female	2,455	49.2
Age (years)	18–39	1,897	38.0
	40–65	3,090	62.0
Habitation	Urban	1,574	31.6
	Rural	3,413	68.4
Educational level	Secondary school or less	3,503	70.2
	Highschool or above	1,484	29.8
Marital status	Single, widowed,divorced	681	13.7
	Married	4,306	86.3
Body weight	Normal or below	3,759	75.4
	Overweight	1,228	24.6
Occupation	Unemployed	425	8.5
	Working (employee or other)	4,562	91.5
Blood pressure	Normal	3,885	77.9
	Elevated	1,102	22.1

## Results

Among the 5,041 survey participants (participation rate 94.1 % [4]), 154 with missing BP data were excluded, leaving 4,987 for analysis (mean age: 43.6 ± 12.8 years; male 2,532 [mean age: 43.4 ± 12.9 years]; female 2,455[mean age 43.9 ± 12.8 years]). There were 466 subjects with migraine, 535 with TTH and 48 with headache on ≥15 days/month. Table 1 shows the distribution of demographic and anthropometric variables in the sample, and Table 2 shows this according to headache type. The prevalence of eBP was 22.1 %(95 % CI: 20.9-23.2 %) overall, 22.9 %(21.2-24.5 %) in males and 21.3 %(19.7-22.9 %) in females.

**Table 2:** Demographic, anthropometric and blood pressure data of the sample according to headache type

Variable	Category	Migraine	Tension-type headache	Headache on ≥ 15 d/m
Gender	Male	150	193	13
	Female	316	342	35
Age (years)	18–39	133	159	6
	40–65	333	376	42
Habitation	Urban	162	209	13
	Rural	304	326	35
Educational level	Secondary school or less	337	374	43
	Highschool or above	129	161	5
Marital status	Single, widowed, divorced	31	56	6
	Married	435	479	42
Body weight	Normal or below	336	387	28
	Overweight	130	148	20
Occupation	Unemployed	40	63	4
	Working (employee, other)	426	472	44
Blood pressure	Normal	345	405	27
	Elevated	121	130	21

d/m: days per month

More detailed analysis of the associations of headache with eBP, by gender, is shown in Table 3. This sets out the estimated 1-year prevalences of migraine, TTH and all causes of headache on ≥15 days/month in participants with eBP or normal BP. Overall (11.0 % vs 8.9 %), and in males (7.9 % vs. 5.3 %), participants with eBP appeared more likely to have migraine, and initial analysis using chi-squared test suggested that these differences were significant (*P* < 0.05). There were no significant differences in TTH. Overall (1.9 % vs 0.7 %), and in females (3.1 % vs 1.0 %), participants with eBP appeared more likely to have headache on ≥15 days/month, and again initial analysis using chi-squared test suggested that these differences were significant (*p* < 0.005). In all cases, however, AORs from multivariate analysis indicated no significance (Table 3).

**Table 3:** 1-year prevalence of headache disorders and associations with elevated blood pressure

Participating sample	Prevalence					
	Migraine		Tension-type headache		Headache on ≥15 days/month	
	*n*	% (95 % CI)	*n*	% (95 % CI)	*n*	% (95 % CI)
All (*N* = 4,987)						
Normal BP (*n* = 3,885)(reference)	345	8.9(8.0-9.8)	405	10.4(9.4-11.4)	27	0.7(0.4-1.0)
Elevated BP (*n* = 1,102)	121	11.0(9.2-12.8)	130	11.8(9.9-13.7)	21	1.9(1.1-2.7)
Total	466	9.3(8.5-10.2)	535	10.7(9.9-11.6)	48	1.0(0.7-1.2)
Chi-squared	4.468		1.687		13.200	
* P*	0.035		0.194		0.000	
OR (95 % CI)	1.3 (1.0-1.6)		1.1(0.9-1.4)		2.8 (1.6-4.9)	
AOR(95 % CI)	1.1(0.9-1.4)		1.0(0.8-1.3)		1.7 (0.9-3.1)	
P (AOR)	0.344		0.954		0.103	
Male (*N* = 2,532)						
Normal BP (*n* = 1,953) (reference)	104	5.3(4.3-6.3)	149	7.6(6.4-8.8)	8	0.4(0.1-0.7)
Elevated BP (*n* = 579)	46	7.9(5.7-10.1)	44	7.6(5.4-9.8)	5	0.9(0.1-1.7)
Total	150	5.9(5.0-6.8)	193	7.6(6.6-8.6)	13	0.5(0.2-0.8)
Chi-squared	5.499		0.001		1.802	
* P*	0.019		0.981		0.180	
OR (95 % CI)	1.5(1.1-2.2)		1.0 (0.7-1.4)		2.1(0.7-6.5)	
AOR (95 % CI)	1.3(0.9-1.9)		0.8(0.6-1.2)		1.4(0.4-4.7)	
P(AOR)	0.150		0.280		0.539	
Female (*N* = 2,455)						
Normal BP (*n* = 1,932) (reference)	241	12.5(11.0-14.0)	256	13.3(11.8-14.8)	19	1.0(0.6-1.4)
Elevated BP (*n* = 523)	75	14.3(11.3-17.3)	86	16.4(13.2-19.6)	16	3.1(1.6-4.6)
Total	316	12.9(11.6-14.2)	342	13.9(12.5-15.3)	35	1.4(0.9-1.9)
Chi-squared	1.278		3.500		12.620	
* P*	0.258		0.061		0.000	
OR (95 % CI)	1.2 (0.9-1.6)		1.3 (1.0-1.7)		3.2 (1.6-6.2)	
AOR (95 % CI)	1.0(0.8-1.4)		1.2 (0.9-1.6)		1.7(0.8-3.6)	
P(AOR)	0.852		0.311		0.143	

*AOR*: adjusted odds ratio; *BP*: blood pressure; *CI*: confidence interval; *OR*: odds ratio

The associations of demographic and anthropometric variables with eBP are shown in Table 4. The strongest associations surviving multivariate analysis were higher age (AOR 3.7) and being overweight (AOR 2.4), and these were seen in both genders. Male gender was also significantly associated, although not very strongly. Lower educational level was a weak but significant factor overall and in females, and urban habitation was similar overall and in males (Table 4).

**Table 4:** Elevated blood pressure and its associations with demographic and anthropometric variables, overall and by gender

Variables	OR(95 %CI)^a^	*P*	AOR(95 %CI)^a^	*P*
All (*N* = 4,987)				
Female gender	0.9(0.8-1.0)	0.183	0.8(0.7-1.0)	0.014
Higher age	4.3(3.6-5.1)	0.000	3.7(3.1-4.4)	0.000
Rural habitation	0.8(0.7-1.0)	0.012	0.8(0.7-0.9)	0.009
Better educated	0.6(0.5-0.7)	0.000	0.7(0.6-0.9)	0.000
Married	2.1(1.7-2.7)	0.000	1.1(0.9-1.5)	0.356
Overweight	2.8(2.4-3.2)	0.000	2.4(2.1-2.8)	0.000
Working	0.9(0.7-1.1)	0.175	0.9(0.7-1.2)	0.507
Male (*N* = 2,532)				
Higher age	3.6(2.8-4.5)	0.000	3.2(2.5-4.0)	0.000
Rural habitation	0.7(0.6-0.9)	0.002	0.7(0.6-0.9)	0.008
Better educated	0.9(0.7-1.1)	0.159	0.9(0.7-1.1)	0.273
Married	2.8(2.0-3.9)	0.000	1.5(1.0-2.2)	0.031
Overweight	2.4(2.0-2.9)	0.000	2.3(1.8-2.8)	0.000
Working	1.1(0.7-1.6)	0.757	1.2(0.8-1.8)	0.492
Female (*N* = 2,455)				
Higher age	5.4(4.1-7.1)	0.000	4.2(3.2-5.6)	0.000
Rural habitation	0.9(0.8-1.2)	0.615	0.9(0.7-1.1)	0.215
Better educated	0.4(0.3-0.5)	0.000	0.5(0.4-0.7)	0.000
Married	1.6(1.1-2.2)	0.007	0.9(0.6-1.2)	0.420
Overweight	3.2(2.6-3.9)	0.000	2.5(2.0-3.1)	0.000
Working	0.7(0.5-1.0)	0.031	0.8(0.6-1.1)	0.219

*AOR*: adjusted odds ratio; *BP*: blood pressure; *CI*: confidence interval; *OR*: odds ratio

^a^References groups for each of the variables are the alternative categories shown in Tables 1 and 2

## Discussion

This large population-based study in Mainland China estimated the prevalence of primary headache disorders [4] and, opportunistically, the adult prevalence of eBP. It had multiple strengths: we recruited a large sample of 5,000 nationwide, inclusive of China’s ethnic, cultural and geographical diversities, promoted data quality by engaging participants in face-to-face interviews and limited bias by achieving a participation rate of well over 90 %. We used a validated questionnaire for headache diagnosis [15]. We measured BP in participants’ own homes, using a standardised procedure. Because of the cross-sectional design of the study, we would not be able to establish causation in any associations that might be observed, but this did not prove to be a limitation. It was, of course, a limitation that we could not collect information about antihypertensive treatments, but we designed the study around that.

The data on headache prevalence have been reported elsewhere [4]. Here we note a prevalence of eBP of 22.1 %, slightly and non-significantly higher in males than females (although multivariate analysis detected a weak but significant association of eBP with male gender). This may be considered to accord with the recently reported adult prevalence of HTN in China of 26.6 % [5], but we discuss this later. First we observe that there were no clear associations between eBP and headache. The weak association between eBP and migraine overall and in males (OR 1.3-1.5) did not survive multivariate analysis. An apparently stronger association with headache on ≥15 days/month (OR 2.1-3.2) was never significant in males and again did not survive multivariate analysis. The confounding effects of age and being overweight – both risk factors for frequent headache [24, 25] and, as we showed, both strongly (and probably causally) associated with eBP were at work here.

In this regard our findings were in line with most of those from previous studies. Cirillo *et al*. [17] found that patients with severe headache had a significantly higher prevalence of HTN, but this study is of limited relevance because of the obvious selection bias. Pietrini *et al*. [18] found that the prevalence of HTN was higher in all headache disorders, including migraine, TTH and “chronic daily headache”, but conducted their study in headache centre outpatients so again there was inevitable selection bias. Neither study employed multivariate adjusted analyses. On the other hand, Waters *et al*. [9] and Rasmussen *et al*. [10] both found no association between headache disorders and HTN, although in the latter study DBP was significantly higher in women with migraine than in women without [10]. Using ambulatory BP monitoring, Kruszewski *et al*. [12] and Gus *et al*. [11] found that active headache was not associated with simultaneous eBP in patients with mild-to-moderate HTN. Some studies suggested inverse relationships. Wiehe *et al*. [15] found that individuals with normal BP complained of migraine more frequently than those with high-normal BP or HTN, while Tzourio *et al*. [14] found migraine in the elderly was associated with lower levels of BP. Goulart *et al*. [13] reported an inverse association between HTN and migraine in men but not in women. An inverse relationship between BP and probability of subsequently developing headache was confirmed in the HUNT study, a large-scale Norwegian population-based cohort survey [16].

In summary, most studies – many of which were well-performed – do not suggest significant associations between headache and HTN, and our findings from this large study do not do so either. Our analyses noted the effects of confounding factors, and illustrate the importance of multivariate analysis, lack of which may at least partially explain the inconsistent results of previous studies. However, it must be remembered that what we observed by recording eBP was not the prevalence of HTN but, essentially, that of untreated or undertreated HTN.

Which brings us back to the issue of public-health concern: that 22.1 % of the Chinese population aged 18–65 years have eBP regardless of any treatment being taken. Associations with higher age, being overweight and, perhaps, lower educational level are unsurprising; the effect of urban habitation, we guess, is mediated through unhealthy living. But the key point is this: while the prevalence of HTN in China may be 26.6 % [3], and HTN is one of the major risk factors for CVDs, which are themselves leading causes of mortality especially in low- and middle-income countries [2], our observation suggests that only one in six affected adults are adequately treated. This ought to be taken very seriously, and the reasons discovered: do the failures lie in diagnosis, health-care coverage, health care itself (wrong treatments) or patient-adherence? Whatever the cause(s), it is clear evidence of unmet health-care need.

## Conclusion

In conclusion, we found no clear-cut associations between eBP and any headache disorder. We did find an alarmingly high prevalence of eBP, recognizing that this signals substantial under-treatment of a serious condition.
